# Reframing RB Tumor Suppressor Dysfunction as a Therapeutic Vulnerability in Cancer

**DOI:** 10.3390/cancers18071175

**Published:** 2026-04-07

**Authors:** Rada Malko, Harlan E. Shannon, Erika A. Dobrota, Keiko E. Kreklau, Lauren K. Stevens, Kyle W. Jackson, M. Reza Saadatzadeh, Pankita H. Pandya, Karen E. Pollok

**Affiliations:** 1 Department of Medical and Molecular Genetics, Indiana University School of Medicine (IUSM), Indianapolis, IN 46202, USA; rmalko@iu.edu (R.M.); phpandya@iu.edu (P.H.P.); 2 Herman B Wells Center for Pediatric Research, Indiana University School of Medicine (IUSM), Indianapolis, IN 46202, USA; heshanno@iu.edu (H.E.S.); erzimmer@iu.edu (E.A.D.); kkreklau@iu.edu (K.E.K.); lasteve@iu.edu (L.K.S.); msaadatz@iu.edu (M.R.S.); 3 Department of Pediatrics, Division of Pediatric Hematology/Oncology, Indiana University School of Medicine (IUSM), Indianapolis, IN 46202, USA; kylwjack@iu.edu; 4 Department of Biochemistry, Molecular Biology, and Pharmacology, Indiana University School of Medicine (IUSM), Indianapolis, IN 46202, USA

**Keywords:** retinoblastoma, therapeutic response, cancer, targeted therapy, cell-cycle

## Abstract

The retinoblastoma (RB) protein was the first tumor suppressor discovered and has been extensively studied for its canonical role in cell-cycle regulation. However, RB has broader noncanonical roles in DNA damage repair, chromosomal stability, apoptosis control, lineage commitment, cell differentiation and broad transcriptional regulation. Historically, *RB* inactivation has been associated with tumorigenesis, as well as resistance to cyclin-dependent kinase 4/6 inhibitors (CDK4/6i), leading to its investigation as a potential predictive biomarker. However, clinical data have not demonstrated that RB function reliably predicts response to CDK4/6i consistently. These discrepancies highlight the need to reconsider RB’s role in therapeutic response, as *RB* loss can promote replication stress, induce chromosomal instability, and lead to transcriptional reprograming, potentially generating context-specific therapeutic vulnerabilities. In this review, we examine the multifaceted biology of RB and evaluate how its loss influences responses to chemotherapy and targeted therapies. We highlight emerging strategies that exploit RB-deficient states using rational monotherapy and combination approaches. Reframing RB dysfunction from a binary biomarker to a driver of exploitable cellular vulnerabilities may inform and expand precision oncology strategies for aggressive and treatment-resistant cancers.

## Simple Summary

Many cancers grow uncontrollably due to the loss of normal cell-cycle checkpoint mechanisms. One key regulator is the retinoblastoma (RB) protein, which acts as a safeguard to control cell proliferation, maintain DNA stability, and guide differentiation and survival. When *RB* is lost or dysfunctional, tumors often behave more aggressively. However, emerging research suggests that *RB* loss may also create specific unique vulnerabilities that can be therapeutically exploited. In this review, we examine the diverse biological roles of RB and discuss how its dysfunction influences responses to chemotherapy and targeted therapies. We highlight evidence showing that *RB* loss may define a distinct therapeutic state, characterized by replication stress, genomic instability, and altered transcriptional programs, that can be targeted using rational combination strategies. A deeper understanding of RB-related pathway dysregulation across cancers may help advance precision oncology approaches for patients with aggressive or treatment-resistant tumors.

## 1. Introduction

**Figure 1:**
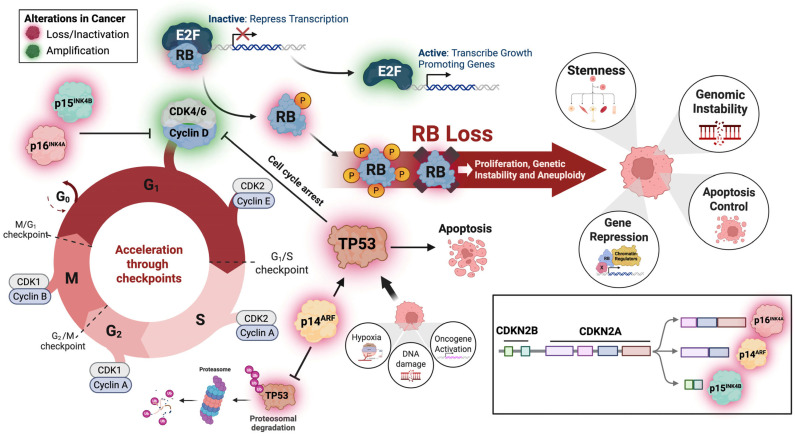
Cell-cycle dysregulation and consequences of *RB* loss in tumorigenesis cells. Cyclins and cyclin-dependent kinases (CDKs) coordinately regulate orderly cell-cycle progression by controlling transitions through key checkpoints. In cancer, amplification or hyperactivation of these complexes accelerates checkpoint progression, leading to uncontrolled proliferation. Concurrently, loss or inactivation of endogenous CDK inhibitors, such as *CDKN2A* (p16^INK4A^ and p14^ARF^), *CDKN2B* (p15^INK4B^), and p21, further removes critical restraints on cell-cycle progression. In response to cellular stress and DNA damage, the TP53 pathway functions to arrest the cell-cycle and facilitate DNA repair or, if damage is irreparable, to induce apoptosis. However, *TP53* is frequently inactivated in cancer, often through amplification of negative regulators such as MDM2 or COPS3, which promote proteasomal degradation of p53. The *CDKN2A*-encoded p14^ARF^ protein normally stabilizes p53 by inhibiting MDM2; thus, loss of p14^ARF^, which is commonly observed in osteosarcoma, leads to p53 destabilization, reduced transcription of downstream targets such as the CDK inhibitor p21, and impaired apoptotic responses. Loss of *RB* function, through hyperphosphorylation or genetic inactivation, releases E2F transcription factors, driving expression of genes that promote proliferation, and genomic instability. All of these alterations enhance cell-cycle progression, suppress apoptosis, and promote tumorigenic phenotypes, including stemness and chromosomal instability. Created in BioRender. Malko, R. (2026) https://BioRender.com/6kbzlq2.

Cancer is a heterogeneous disease marked by unregulated proliferation. Accelerated cell division drives genomic instability and promotes rapid accumulation of genetic aberrations causing tumorigenesis, progression, and drug resistance. Among numerous dysfunctional cancer pathways, retinoblastoma (*RB*) and *TP53* are the most frequently altered tumor suppressor genes, occurring in approximately 73% and 69% of patients, respectively [1]. Large-scale sequencing studies from 200,000 solid tumors found that over 25% harbor genetic alterations in key cell-cycle regulators, including *CCND1-3*, *CCNE1*, *CDK4/6*, *CDKN2A/B*, *SMARCB1*, *AR*, *ERS1*, or *RB*, as summarized in Figure 1 [2]. The cell-cycle is a continuous process controlling proper replication and cell division, regulated by various checkpoint mechanisms. The first critical checkpoint at G1/S is controlled by the RB-E2F-CDK4/6 pathway. When RB is hypophosphorylated, it binds E2F keeping it inactivated, repressing genes needed for S-phase entry. This checkpoint is also controlled by P53/P21, which inhibits cyclin-dependent kinase (CDK) activity to allow for DNA damage repair or apoptosis induction.

In cancer, cell-cycle regulators are frequently inactivated by *RB* loss or RB hyperphosphorylation, E2F overexpression, *CDK4/6* or *CCND1-3* amplification, P53 dysregulation, or inactivation of endogenous CDK inhibitors like *CDKN2A/B*. All of these alterations converge on E2F-mediated transcription, a central driver of uncontrolled cell-cycle progression. These dysregulations can be targeted using CDK4/6 inhibitors (CDK4/6i), which suppress RB phosphorylation and enforce G1 arrest. While functional RB has historically been considered a biomarker for CDK4/6i sensitivity, these relationships may be more complex. Interestingly, *RB* deletion or inactivation is typically mutually exclusive with other related CDK4/6 pathway deregulation, since both lead to similar downstream effects. However, a single copy loss of *RB* may co-occur with CDK4/6 pathway alterations, especially in advanced or therapy-resistant tumors [3]. These observations suggest that tumor cells may derive benefit from CDK4/6 inhibition independent of RB status, potentially through noncanonical RB-mediated pathways that influence genomic stability.

RB has diverse functions including DNA repair, apoptosis regulation, and broad transcriptional regulation, all of which influence therapeutic response. Thus, loss of *RB* may promote oncogenic replication stress and disrupt DNA repair pathways, increasing mutational burden of cells. Ultimately, the increasing reliance of RB-deficient cells on residual stress response mechanisms may sensitize them to DNA-damaging agents and targeted therapies. The additional stress may accelerate replication stress beyond tolerable thresholds and ultimately induce cancer cell death. In addition, RB-dependent regulation of apoptosis, differentiation, and chromosome maintenance may also affect cellular stress responses. The broad roles of RB underscore how RB dysfunction, frequently observed in cancer, creates distinct therapeutic vulnerabilities that may be exploited by rational treatment strategies.

The scope of this review is to examine the various roles of RB and how its loss or dysregulation influences critical cellular pathways. Given that many cancers have some form of RB pathway dysregulation, often in settings with limited therapeutic options and poor survival, exploiting cellular vulnerabilities represents a promising opportunity. In this context, *RB* loss may define a distinct state that reshapes treatment logic from a single-agent prediction to rational combination strategies. Understanding how RB status affects multi-agent therapy response, rather than resistance alone, is central to its clinical utility. Thus, the purpose of this review is to first examine the utility of CDK4/6i to limit accelerated cell proliferation. Next, the review will explore the many functions of RB and the potential pathways affected to strategize utility of targeted therapies. Finally, we consider how biomarkers like RB have influenced the efficacy of cancer therapeutics to date in preclinical and clinical studies.

## 2. RB Protein

### 2.1. The RB Family Proteins: Composition and Functional Roles in Cell-Cycle Regulation

RB is a member of the pocket protein family, which includes RBL1 (p107) and RBL2 (p130). These proteins have evolutionarily conserved similarities, most notably the A and B pocket domains. This shared domain mediates protein interactions with E2F transcription factors and other regulatory proteins controlling transcription. RB is the primary regulator of the G1/S transition, where it suppresses E2F1-5 target genes required for DNA synthesis and cell division. In contrast, p107 and p130 are associated with the regulation of quiescence and differentiation, binding E2F4-5 to form the dimerization partner, RB-like, E2F, and the multi-vulval class B (DREAM) complex [4]. Despite overall structural similarity, RB family members have distinct biological functions. Both p107 and p130 contain B-domain insertions and larger spacer regions compared to RB, which have been associated with increased protein stability [5]. In addition, the conserved N-terminal domains of p107 and p130 enable direct binding and inhibition of CDKs, even in the absence of phosphorylation [6]. In contrast, RB uniquely contains an E2F1 docking site and a C-terminal region that can be occupied by cyclin/CDKs or protein phosphatase 1, facilitating direct repression of transcription programs that drive S-phase entry [5]. Thus, while all three proteins contribute to cell-cycle control, they differ in structure, expression patterns, and binding partners.

Functional redundancy of the RB family members allows for compensation when one pathway is disrupted or nonfunctional. RB/E2F and DREAM complexes recruit chromatin modifiers and repress overlapping sets of genes, although their mechanisms of action are different [7]. The DREAM complexes bind promoter regions of target genes to actively repress transcription using corepressors like histone deacetylases (HDACs) and ATPase subunits BRM/BRG1 of the SWI/SNF chromatin-remodeling complexes [8]. In addition to E2F target genes, the DREAM complex interacts with DNA at cell-cycle gene homology (CHR) and CHR-like element promoter regions, altering gene expression during both G1-S and G2-M transitions [9]. While p130 and p107 are less frequently mutated in cancer compared to RB, they have similar roles in cell-cycle regulation. These roles may be particularly crucial in the context of *RB* loss, which is inactivated through multiple mechanisms, including genomic, epigenetic, and oncogenic alterations.

### 2.2. Heterogeneity of RB

RB pathway alterations in cancer are highly heterogeneous. They can arise from genetic, epigenetic, and post-translational modifications, ultimately disrupting RB function or *RB* gene expression. At the protein level, RB can be lost through gene deletions, loss of heterozygosity, mutations, and even transcriptional silencing. RB inactivation also frequently occurs due to upstream pathway dysregulation that leads to persistent RB hyperphosphorylation. Examples include amplification of CDK4/6 or cyclins, as well as loss of endogenous CDK inhibitors. Post-translational modifications, particularly phosphorylation, regulate RB activity even in the absence of genetic alterations. A pan-cancer molecular analysis of RB-related pathways demonstrated frequent single-copy loss of *RB* in solid tumors, often co-occurring with alterations in CDKs, cyclins, or *CDKN2A* [3]. In this study, *RB* was deleted or mutated (7%), *CDKN2A/B* was deleted (25%), and *CDK4/6* or *CCND1-3* was amplified (13.1%), highlighting the importance of indirect RB pathway suppression.

The effect of RB alterations may depend on the cancer subtype and remains an area of ongoing research. There is a high prevalence of *RB* loss or inactivation in hereditary retinoblastoma, small cell lung cancer, osteosarcoma, prostate cancer, and bladder cancer. In hereditary retinoblastoma, *RB* inactivation is caused by insertions/deletions and single nucleotide substitutions (80%) or chromosomal rearrangements (20%) [10]. Over 900 different *RB* mutations have been associated with hereditary retinoblastoma. Different mutations result in variable penetrance and pathogenicity, highlighting the substantial heterogeneity that exists even within one specific tumor type [11]. Epigenetic silencing through promoter hypermethylation, another mechanism of *RB* inactivation, is frequently found in hereditary retinoblastoma, breast, colorectal, lung, and prostate cancers [12]. RB exists along a spectrum ranging from fully functional to completely inactivated, depending on the alteration and the available cellular compensatory mechanisms. Collectively, this heterogeneity underscores the complexity of the RB pathway dysregulation and its implications on biology and therapeutic response.

## 3. Targeting Cell-Cycle Dysregulation

Innovations in precision oncology have identified cell-cycle dysregulation as a hallmark of cancer and an actionable therapeutic signature. The clinical utility of CDK4/6i, including palbociclib, abemaciclib, and ribociclib, was demonstrated by their FDA approvals between 2015 and 2017 for the treatment of RB-proficient breast cancer in combination with hormone therapies [13,14,15]. CDK4/6i canonically block CDK4/6-mediated RB phosphorylation, keeping transcription factors like E2F inactive and repressing proliferation. However, cyclin–CDK complexes can phosphorylate many other substrates, like FOXM1 to promote G2-M transition, c-MYC causing oncogene activation and RUNX2 repressing osteogenic differentiation. Additionally, independent of its kinase activity, CDK6 can directly interact with transcription factors, including c-JUN, RUNX1 and STAT3 [16]. Together, these findings suggest that CDK4/6i may exert anti-tumor effects even in RB-deficient cells through alternative noncanonical anti-tumor pathways. This highlights the need for additional investigation of tumors with reduced or absent RB expression due to loss-of-function mutations, loss of heterozygosity, or homozygous *RB* gene deletions.

In addition to the broad anti-tumor effect of these CDK4/6i, it is important to note they are not made equal. Each drug differs in its potency, toxicity, and even target selectivity. For example, palbociclib and ribociclib are administered once daily due to their long half-life (30 h), while abemaciclib requires twice daily dosing due to its shorter half-life (18 h) [17]. While abemaciclib has gastrointestinal toxicities, it lacks the hematological toxicity associated with palbociclib and ribociclib and can be given continuously. However, treatment breaks are needed for palbociclib and ribociclib to allow for bone marrow cell recovery. Additionally, abemaciclib is lipophilic and can penetrate tissues and the blood–brain barrier more effectively [18]. While palbociclib and ribociclib are selective CDK4/6 inhibitors, abemaciclib has broader kinase activity inhibiting CDK1, CDK2, CDK5, CDK7, and CDK9 at higher concentrations. The differences between these FDA-approved CDK4/6i may affect overall drug efficacy and have context-dependent utility.

Less selective, first-generation pan-CDK inhibitors, like flavopiridol, were discontinued from clinical development due to dose-limiting toxicities from a lack of target specificity. Other non-FDA-approved CDK4/6i, like lerociclib and dalpiciclib, are approved in China for the treatment of advanced breast cancer. Distinct from the others, trilaciclib is the only FDA-approved CDK4/6i formulated for intravenous administration, not for anti-tumor activity but to mitigate chemotherapy-induced hematological toxicities in patients with small cell lung cancer [19]. Despite the overall success of CDK4/6i, reliable biomarkers of sensitivity and resistance remain limited. Although RB-proficient status is often considered a prerequisite for anti-tumor response, it may not be an absolute predictor since no universally accepted biomarker has yet been established. While the efficacy of CDK4/6i has reshaped the treatment of breast cancer, it remains unclear if CDK4/6i can achieve similar benefits in other malignancies or in RB dysregulated tumors, warranting further research.

### 3.1. Questioning RB’s Reliability as a Therapeutic Marker

Understanding how RB status affects therapeutic response is critical to determine biomarkers of resistance and sensitivity to CDK4/6i. To date, functional RB status has often guided clinical decisions to use CDK4/6i, since multiple preclinical studies have suggested that RB-proficient status is a requirement for efficacy [20,21,22,23,24]. For instance, studies conducted in 2004 by Fry et al. demonstrated that palbociclib had no antiproliferative effect in RB-deficient breast and non-small cell lung cancer cell lines, requiring concentrations over 50 times the level needed for proficient cell lines [21]. Furthermore, palbociclib was found to be inactive against RB-deficient breast carcinoma tumor xenografts. However, subsequent studies have yielded more nuanced results. In 2010, Dean et al. similarly found reduced sensitivity to palbociclib in RB-deficient settings but, when *RB* was knocked out, inconsistent results were reported, indicating a subtle but notable discrepancy [24]. For instance, palbociclib treatment significantly reduced BrdU incorporation regardless of RB status, suggesting RB may not be required for an antiproliferative response. Additionally, they found that E2F increased the levels of RB isoform p107 only in palbociclib-treated RB-deficient cells, suggesting that E2F overexpression may be more important than *RB* loss for sensitivity to CDK4/6i.

These early reports of variable responses to CDK4/6i highlight the inherent complexity of proliferative control in cancer. This variability likely reflects cell-specific molecular contexts that influence sensitivity to agents such as palbociclib. Under canonical conditions, mitogenic signaling activates CCND-CDK4/6 complexes, leading to RB phosphorylation, release of E2F transcription factors, and induction of S-phase gene expression. CDK4/6i block this pathway, resulting in G1 arrest. Historically, RB has been considered a key determinant of CDK4/6i efficacy, given its role as a primary downstream target. However, emerging evidence indicates that CDK4/6i activity is not exclusively dependent on RB status. Alternative mechanisms such as CCNE-CDK2-mediated RB phosphorylation, E2F overexpression, and compensatory functions of RB family members (p107 and p130) can sustain cell-cycle progression or modulate drug response. These redundancies and parallel regulatory pathways suggest that RB status alone may be insufficient as a standalone predictive biomarker for patient stratification and CDK4/6i response.

Several preclinical studies reported p16 (*CDKN2A*) overexpression in the RB-deficient cell lines, which may be the underlying reason for palbociclib resistance, rather than exclusively RB status [23,25]. However, preclinical studies have focused on the efficacy of CDK4/6i in the context of *RB* loss, CCND/E amplifications, and p16 loss as determinants of response to CDK4/6 inhibition; these alterations alone do not fully predict response. Multiple compensatory pathways may bypass CDK4/6i, complicating the efficacy observed between tumors that are RB-proficient or RB-deficient. Importantly, clinical data mirrors these findings. In the PALOMA-2 breast cancer trial testing palbociclib, expression of RB, CDK2/4/6, CDKN2A, or CCND1/E at the mRNA or protein levels was not predictive of sensitivity to CDK4/6i therapy [25]. Additional biomarker analysis from the same researchers used data from the PALOMA-2 study to look more closely at whether RB expression could predict response to palbociclib. They reported no difference in progression-free survival (PFS) between high and low expressing RB tumors [26]. These data suggest that RB status may not predict response to palbociclib and alternative mechanisms could also be driving resistance to CDK4/6i.

These discrepancies between studies likely reflect underlying heterogeneity in RB pathway alterations, experimental systems, tumor subtype, and molecular contexts. Differences between in vitro models, in vivo studies, and clinical trials may contribute to additional variability in observed responses to CDK4/6 inhibition. In addition, tissue-specific genetic markers of *RB*, *TP53*, *E2F*, and *CDKN2A/B* and activation of cyclin/CDK pathways significantly influence therapeutic outcomes. Together, these factors provide a framework for understanding the variable predictive value of RB status across different tumor types and molecular contexts.

### 3.2. RB-Independent CDK4/6i Efficacy

Identifying RB-independent mechanisms that drive anti-tumorigenic responses is critical for broadening the utility of CDK4/6i, particularly in patient populations with dysregulated RB or resistance development. Moreover, the complexity of variable responses observed with CDK4/6i highlights the need for continued research, particularly in RB-deficient contexts where knowledge is limited. RB has long been recognized as the canonical downstream target of CDK4/6i. In RB-proficient settings, CDK4/6 inhibition maintains RB in an active, hypophosphorylated state, suppressing E2F-driven transcription and resulting in cell-cycle arrest. In contrast, in RB-deficient contexts, CDK4/6i may act through alternative pathways, including transcriptional modification, DNA repair processes, and cellular differentiation, highlighting their role beyond the canonical RB–E2F axis regulation [16].

CDK4/6 complexes have been shown to phosphorylate and activate FOXM1, independent of RB proteins, leading to reduced reactive oxygen species, protection from senescence, and increased proliferation [27]. Similarly, CCND1 plays a role in DNA repair, as it is recruited via BRCA to sites of DNA damage and supports homologous recombination (HR), with effects conserved in RB-deficient cancer cells [28]. The cyclin/CDK complexes also phosphorylate and repress differentiation of various cell types, including cardiomyocytes via GATA4, muscle cells via MEF2, neurons via NGN2, and bone cells via RUNX2 [16]. Additionally, CDK6 can directly interact with transcription factors c-JUN and STAT3 to stimulate angiogenesis and proliferation in haemopoietic malignancies such as B-cell acute lymphoblastic leukemia [29]. Collectively, these functions may contribute to RB-independent efficacy of CDK4/6i.

Moreover, functional redundancies among RB-family proteins exist. Over 85% of the same genes are regulated by the P53-p21-RB and the related P53-p21-DREAM pathways [4]. Because of this, RB dysregulated tumors may respond to CDK4/6i through compensatory proteins like p107 and p130 [30]. Since *RB* genetic alterations are found in a substantial fraction of cancer patients and RB dysregulation has been proposed as a mechanism of acquired resistance, it is essential to evaluate whether CDK4/6i are effective in RB-deficient contexts. Emerging data demonstrate that RB functions extend beyond cell-cycle control to include genomic stability, induction of apoptosis, and cellular stemness [31,32,33,34,35,36], further supporting the concept that RB dysregulation may impact broader cellular processes and present a therapeutic vulnerability that can be exploited.

## 4. RB Biology and Its Multifaceted Role in Cancer

### 4.1. Role of RB in Apoptosis

As previously mentioned, RB has multifaceted roles that expand beyond cell-cycle regulation. For example, RB has been implicated in both the induction and inhibition of apoptosis, with effects depending on the cell type involved. Apoptosis is often dysregulated in cancer, and many therapeutic strategies aim to induce extrinsic (death receptor) and intrinsic (mitochondrial) pathways. Both pathways converge on executioner caspase activation and ultimately DNA fragmentation and phagocytosis. However, RB has a complicated role in apoptosis regulation depending on the cell type and context. Early developmental studies reported that, while RB-null embryos underwent apoptosis, this effect was largely abrogated if both *RB* and *TP53* were lost [35]. Studies in mice with slow-growing, apoptosis-prone brain tumors confirmed that *RB* loss was associated with apoptosis but also demonstrated that loss of E2F reduced apoptosis by 80%, indicating a role in P53-dependent death, which may be explained by transcriptional regulation of pro-apoptotic factors [37]. Thus, when *RB* is lost, apoptosis may be dependent on alternate pathways such as E2F and P53.

**Figure 2:**
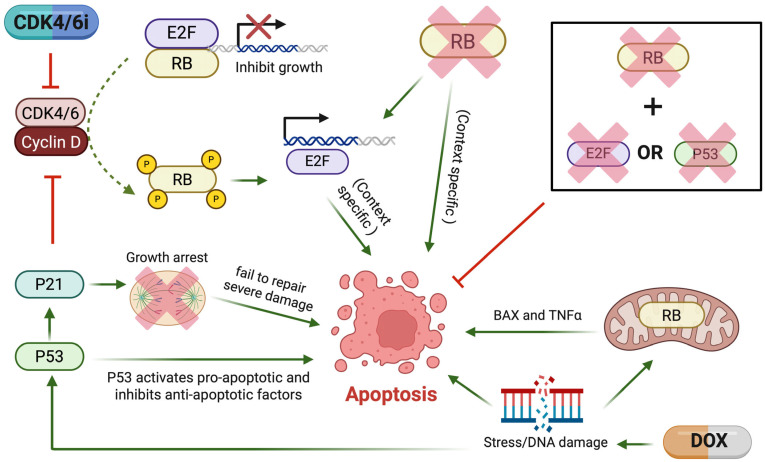
Context-dependent roles of RB in apoptosis regulation. CCND-CDK4/6 complexes phosphorylate RB, leading to release of E2F and promotion of cell proliferation. *RB* loss can induce apoptosis, which is reduced by concurrent loss of p53 or E2F. In cancer, co-alterations of these pathways may favor proliferation over apoptosis. Under conditions of cellular stress such as exposure to doxorubicin (DOX), E2F can induce transcription of pro-apoptotic factors. DNA may also trigger apoptosis directly or through RB-mediated mitochondrial activation of BAX and TNFα. In parallel, P53 is activated in response to DNA damage to promote repair; if damage is irreparable, cells undergo apoptosis. Together, these pathways illustrate the context-dependent roles of RB in regulating both cell survival and cell death. Created in BioRender. Malko, R. (2026) https://BioRender.com/el19le1.

Since P53 and RB dysregulation frequently occur early in cancer development to promote unregulated growth, co-occurring alterations may be a mechanism of apoptosis evasion in cancer. In contrast, studies have also demonstrated that genotoxic agents, like doxorubicin (DOX), promote RB/E2F-dependent transcriptional activation of pro-apoptotic genes and repression of cell-cycle genes. Notably, DOX induced DNA damage in both glioblastoma (P53 null) and osteosarcoma (wild-type P53 and RB) cell lines, suggesting that this apoptotic effect is independent of P53 [38]. Additional studies have found that, in response to cellular stress, RB in the mitochondria directly binds and activates pro-apoptotic factors, including Bax and TNF-α [34]. These findings, summarized in Figure 2, highlight that RB-dependent effects on apoptosis are influenced by key determinants, such as P53 status, E2F activity, cellular stress, and specific molecular perturbations. Thus, several factors ultimately determine whether RB signaling promotes or inhibits apoptosis.

### 4.2. RB in Stemness and Differentiation

During early development, a time of high cell plasticity, RB proteins are required for proper embryonic stem cell (ESC) differentiation and survival [39]. In contrast, *RB* loss in cancer has been frequently associated with dedifferentiation and the acquisition of stem-like features, both traits often linked to poor prognosis. Interestingly, these stem-like states potentially resensitize cells to apoptosis responses, paralleling those seen in early development. While the origin of cancer stem cells (CSCs) is unclear, the presence of tumoral CSCs is important to consider in cancer therapy, as their properties contribute to disease aggressiveness, therapy resistance, and relapse. Mechanistically, Chang et al. demonstrated that the RB/E2F/WNT pathway regulates self-renewal, chemoresistance, and invasiveness of CSCs in breast cancer, pancreatic cancer, and fibroblasts [40]. In these models, E2F induces and RB/RBL2 reduces WNT ligand expression, with WNT upregulation promoting epithelial–mesenchymal transition (EMT) through transcription factors ZEB and SNAI. Interestingly, palbociclib suppressed WNT/β-catenin signaling, thereby reducing CSC proliferation and dedifferentiation. Similarly, Gu et al. reported that *RB* loss confers stem-like properties through E-cadherin suppression of ZEB and identified several CDK inhibitors that effectively suppress ZEB [41]. These studies suggest that *RB* loss may increase EMT and cancer stem-like properties, but this may be context-dependent.

RB also plays a critical role in differentiation and lineage commitment across several biological contexts by interacting with lineage-specific transcription factors, such as MyoD for myogenesis, RUNX2 for osteogenesis, and EBP for adipogenesis [36,42,43]. Functional studies using inducible expression of a mutant SV40 T antigen (T_121_) vector to inactivate RB family proteins inhibited proliferation and differentiation, resulting in G2/M arrest and cell death in human ESCs [40]. Additionally, RB can directly bind and repress pluripotency genes like *Sox2* and *Oct4*, and loss of *RB* can reprogram differentiated cells into a more pluripotent state to initiate tumorigenesis [44]. Some cancers may develop due to malignant transformation of stem cells, and the subtype is dependent on the point along the cell differentiation trajectory where mutations in genes like *RB* are acquired [45]. Together, these findings underscore RB’s crucial role in stem-like properties and differentiation pathways and highlight the need for continued investigation of how RB dysregulation may affect normal pathways associated with stemness and differentiation.

### 4.3. RB and Maintaining Genomic Stability

Tumorigenesis increases with genomic instability due to the accelerated frequency of acquired mutations. This instability leads to defective DNA repair, improper chromosomal segregation, and loss of checkpoint control pathways [32]. Notably, RB is implicated in the two primary DNA double-strand break (DSB) repair pathways. RB recruits BRG1 chromatin remodelers aiding in HR and interacts with KU70/80 to facilitate non-homologous end joining (NHEJ) repair [33,46]. As a result, RB-deficient tumors may be vulnerable to therapies like PARP inhibitors (PARPi) that exploit their defective DNA repair pathways. RB also recruits HDACs and DNA methyltransferases to regions of repetitive DNA, which makes up over 50% of the human genome, maintaining a silenced heterochromatic state. Loss of these regions causes defective chromosome segregation, illegitimate recombination events, and increases chromosomal instability (CIN) [47]. For instance, one study found that the RB/E2F complex recruits EZH2 methyltransferase complex to mediate H3K27me3 silencing of repetitive genomic regions, and loss resulted in lymphoma, suggesting a critical role in cancer development [47]. Thus, *RB* loss affects chromosomal structure and integrity, leading to instability.

Additionally, RB is important for mitotic fidelity and proper chromosomal segregation. *RB* loss may promote aneuploidy since it is involved in chromosomal cohesion, spindle formation checkpoints, and kinetochore assembly [31,48]. It is important to review that typical human cells are diploid with one set of chromosomes from each parent. The amount of DNA changes throughout the cell-cycle since cells grow during G1, DNA is replicated after S-phase and cell division occurs following mitosis. In cancer, errors in ploidy and DNA content result from dysregulated checkpoints and improper progression. Conklin et al. found that, when all RB family members are inactivated in human ESCs, they exhibit abnormal genome doubling, deviations from normal ploidy, and extensive aneuploidy as demonstrated by karyotyping analysis, indicative of irregular genomic content [39]. *RB* loss is also associated with chromothripsis, catastrophic chromosome shattering and reassembly events, likely arising from segregation errors, micronuclei formation, and telomere crisis [49]. Therefore, RB maintains genomic stability and its loss drives CIN through diverse mechanisms that may create potential vulnerabilities for therapeutic intervention. These multifaceted roles underscore why RB status could serve as both a biomarker and a therapeutic target and should be further investigated.

## 5. Therapeutic Implications of RB Status

**Figure 3:**
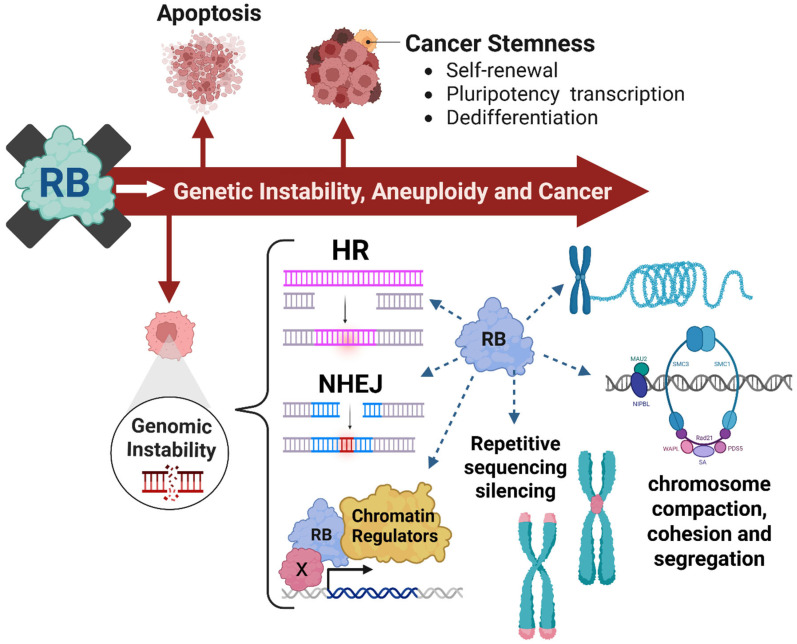
Therapeutic vulnerabilities created by *RB* loss promoting genetic instability, aneuploidy, and tumorigenesis. Beyond its canonical role in repressing E2F-mediated transcription, RB functions as a central regulator of many cellular processes. It modulates apoptotic pathways in both the mitochondria and cytoplasm. RB also controls genomic stability through regulation of DNA repair, transcriptional control, and chromatin organization. Additionally, it contributes to the stability and maintenance of telomere and centromere structure integrity, chromosome compaction, and cohesion functions. RB also influences cancer stemness by controlling self-renewal, differentiation, and pluripotency of various cell types. Created in BioRender. Malko, R. (2026) https://BioRender.com/13vjg8s.

The RB pathway is disrupted in a wide range of human cancers and influences numerous cellular processes in a context-dependent manner illustrated in Figure 3. Even with its diverse regulatory functions, RB status is not routinely used as a clinical biomarker of therapeutic response. While studies in this area are limited, there are many ongoing efforts focused on aligning genetic signatures with targeted therapies to develop predictive biomarkers of treatment response. Currently, CDK4/6i are frequently used to target cell-cycle dysregulation; however, RB status is also important in inducing replication stress and altering sensitivity to mitotic checkpoint inhibitors, PARPi, and bromodomain and extra terminal domain inhibitors (BETi).

However, a major challenge in assessing RB status clinically using traditional immunohistochemistry is that these methods may miss functional loss in cases where RB protein is mutated or levels fluctuate during proliferation [50]. Moreover, *RB* can be inactivated by several mechanisms, including gene mutations or deletions, epigenetic silencing, or post-translational modifications, prompting the development of other methods, like transcriptomics, to identify *RB* loss signatures. Beyond protein expression-based approaches, several alternative strategies have been proposed to more accurately assess functional RB status. These include gene expression signatures of *RB* loss, assessment of *RB* promoter hypermethylation, and multi-omics approaches that integrate genomic, transcriptomic and proteomic data. Assays evaluating E2F target activity or cell-cycle proteins also provide insight into RB functional status, as RB may be inactivated through other mechanisms like hyperphosphorylation or through amplification of related proteins like cyclins or CDKs. Together, multiple approaches may offer a more comprehensive assessment of RB function and improve its utility as a biomarker of targeted therapies, including CDK4/6i.

Despite recent molecular advancements, significant challenges remain in identifying and translating RB-associated biomarkers into clinical practice. These limitations include a lack of standardized assays to measure RB dysfunction and limited prospective clinical validation. The variability in detection methods complicates the interpretation of RB status across studies. Additionally, the heterogeneity of RB pathway alterations and co-occurring genetic events make interpretation of biomarker signatures difficult. Notably, RB loss does not result in uniform vulnerability, as responses depend on tumor-specific molecular characteristics (e.g., P53, MYC, and E2F) and the treatment being used. Depending on the nature of RB dysregulation, distinct exploitable pathways may guide different rational combination strategies. Importantly, the impact of *RB* loss is highly context-dependent and varies across tumor types.

While RB deficiency may create therapeutic vulnerabilities in certain cancers, these effects are not universal and can be influenced by tumor-specific biology, additional genetic alterations, and the type of RB dysfunction acquired. To address these challenges, future efforts should focus on multicenter validation studies and biomarker-driven clinical trials with stratified patient cohorts. Integration of molecular and clinical parameters will improve the utility of RB-associated biomarkers for predicting efficacy across cancer therapies. In the next sections, we review current evidence of RB as a biomarker for chemotherapy and targeted therapies including CDK4/6i and other more novel RB-informed therapeutic strategies. Importantly, this does not suggest that *RB* loss is beneficial but rather reflects a context-dependent vulnerability that may be exploited.

### 5.1. Impact of RB Status on Chemotherapy Response and Maintaining Genomic Stabiltiy

The influence of RB status on chemotherapy response remains incompletely understood. Growing evidence suggests that, while *RB* is a frequently lost tumor suppressor in cancer, driving progression, it may create therapeutic vulnerabilities that can be exploited under specific conditions. For instance, genotoxic therapies may be one mechanism, due to enhanced replication stress and altered DNA repair capacity upon RB dysfunction. Chemotherapy consists of many drug classes, with different mechanisms of action, with the goal to reduce cell division, induce DNA damage, and promote cell death. DOX has served as a first-line treatment option in aggressive cancers. As an effective DNA damaging agent, DOX inhibits topoisomerase II, an enzyme that prevents DNA unwinding, leading to supercoiling and strand breakage [51,52,53,54]. It has broad effects cellularly, affecting reactive oxygen species generation, iron chelation, ceramide level modulation, and immunogenicity, all of which increase its cytotoxic efficacy [55]. As a result, DOX has remained the treatment of choice for many aggressive and metastatic cancers, like soft tissue sarcomas (STS), since clinical trials testing combinations with other chemotherapies have failed to improve survival outcomes relative to DOX alone, as summarized in section A of Table 1 [56,57,58].

Combination chemotherapy regimens are a mainstay of cancer treatment. Combinations may include antimetabolites (e.g., methotrexate, 5-Fluorouracil (5-FU), and gemcitabine), which mimic endogenous nucleotides and disrupt proper DNA synthesis and replication, taxanes (e.g., docetaxel), which alter microtubule formation and increase mitotic catastrophe, and alkylating agents (e.g., ifosfamide, palifosfamide, and cisplatin), which induce DNA cross-links that impair replication and transcription. Notably, no agent has demonstrated superior efficacy or tolerability over DOX-based therapy. However, even though survival has improved for many patients, survival outcomes remain poor for aggressive and metastatic sarcomas (<10%) [59]. Additionally, chemotherapy sensitivity varies by cancer subtype and patient populations, reflecting tumor heterogeneity and the need for biomarker characterization [60].

RB status has emerged as a potential biomarker due to its various roles in cellular processes. *RB* loss accelerates cell-cycle progression and replication stress, potentially sensitizing tumors to DNA-damaging agents. Genome-wide CRISPR screens have identified that RAD18, a mediator of HR DNA damage response, is a marker of DOX resistance in osteosarcoma. These studies additionally found that *RB* loss restored chemotherapy sensitivity [61]. Interestingly, RB and RAD18 are both involved in HR; thus, disruption of either pathway may enhance DOX response. Moreover, *RAD18* is transcriptionally activated by E2F3, which highlights the similarity between these independent pathways [62]. Additional studies have reported that high HIF-1α expression correlates with decreased response to chemoradiotherapy and high γH2AX levels predicted favorable outcomes [63]. Biomarker investigations in head and neck cancers found that higher mRNA expression levels of *TP53*, *Bcl-2*, *VEGF*, *ERCC1*, and *XPA* were associated with higher sensitivity to chemotherapies like 5-FU and cisplatin [64]. They also found that higher levels of *RB* and *E2F* were not significant. These findings underscore the need for biomarker-driven stratification, particularly for patients with advanced or metastatic disease.

The potential of RB as a biomarker of chemotherapy sensitivity has been reviewed previously in detail [65]. Here, we highlight a few examples in which *RB* loss has been associated with an increased therapeutic response. In estrogen receptor (ER)-negative breast cancer, while *RB* loss signatures correlated with poor prognosis, they were associated with increased sensitivity to chemotherapy [66]. Witkiewicz et al. analyzed gene expression profiles of patients that were treated with 5-FU, cyclophosphamide, and DOX, and additionally found that *RB* loss improved response to neoadjuvant chemotherapy in both ER-positive and -negative groups [67]. Similarly, Robinson et al. found that RB deficiency was associated with increased response to radiation therapy and chemotherapy in triple-negative breast cancer (TNBC) [68]. An ongoing retrospective clinical trial (NCT01514565) is investigating whether tumor suppressors, such as RB, correlate with chemotherapy sensitivity in TNBC. To date, clinical studies have identified that *RB* loss is the only predictive factor of improved response to treatment with methotrexate, 5-FU, and cyclophosphamide, with RB-deficient tumors showing more DSBs and reduced proliferation after treatment [69].

Interestingly, while *RB* loss was associated with better prognosis in patients receiving chemotherapy, it led to worse outcomes in those receiving hormone therapy, emphasizing the context-dependent role of RB and the importance of therapy-specific vulnerabilities. These findings highlight the need to investigate RB status more broadly across tumor types. An ongoing clinical trial (NCT00580385) aims to evaluate therapeutic responses across various solid tumors, to identify predictive biomarkers of sensitivity and resistance, including *RB* alterations [70]. Together, these findings, summarized in Section B of Table 1, suggest that RB status needs additional investigation across various cancer types, since it may be a biomarker of chemotherapy sensitivity.

**Table 1:** Biomarkers of therapeutic response to chemotherapy and targeted therapies in cancers.

Groups	Eligibility	Results	Ref.
**A. Chemotherapy Response in Sarcomas**			
Treatment groups: DOX v DOX + palifosfamide	Patients with untreated metastatic STS (NCT01168791)	No significant difference between groups. Median PFS was 5.2 and 6 months for DOX alone and combination therapy, respectively	[56]
Treatment groups: DOX v gemcitabine + docetaxel	Previously untreated advanced or metastatic STS (ISRCTN07742377)	Median PFS was 23.3 and 23.7 weeks for DOX and combination, respectively.	[57]
Treatment groups: DOX v DOX + ifosfamide	Patients with advanced or metastatic STS (NCT00061984)	No difference in overall survival in groups using DOX alone (12.8 months) or its combination (14.3 months).	[58]
**B. Identification of Biomarkers of Chemotherapy Sensitivity**			
Identify biomarkers in sarcoma patients treated with radiation + DOX + ifosfamide	Patients with aggressive STS treated with chemoradiotherapy (NCT03651375)	High expression of HIF-1α correlated with poor response while high γH2AX correlated with favorable response to therapy.	[63]
Identify biomarkers that predict response to chemotherapy	64 head and neck cancer patients (ages 31–79) treated with cisplatin + 5-FU and radiation	High mRNA expression of *TP53*, *BCL-2*, *VEGF*, *ERCC1*, *XPA*, *MDR1* was associated with chemotherapy sensitivity (RB *p*-value = 0.13).	[64]
Determine is *RB* loss increases sensitivity to antimetabolites	518 patients (ages 25–89) who had breast cancer surgical resection	Loss of *RB* was the only predictive factor of improved response to cyclophosphamide, methotrexate, and 5-FU chemotherapy	[69]
Identify tumors that respond/fail to respond to various therapies	Patients with bone or solid tumors (NCT00580385)	Determine resistance mechanisms after methotrexate. Also test sensitivity in vitro to therapy and correlate it with molecular studies.	[70]
**C. Targeted Therapy Response and Biomarkers in various cancers**			
Treatment groups: palbociclib + letrozole vs letrozole	ER+/HER2- patients from the PALOMA-2 trial (NCT01740427)	Found that expression levels of genes in the RB/Cyclin/CDK pathway didn’t correlate with therapeutic efficacy.	[71]
RB positive or CDK4/6 altered tumors treated with palbociclib	Breast cancer and solid tumor patients from (NCT01037790)	Biomarker study found that neither RB, Ki-67, and p16 were significantly associated with benefit in all groups.	[72]
Sarcomas treated with palbociclib targeted therapy	Sarcomas with high CDK4 and low/normal p16 expression (mRNA) (NCT01740427)	Biomarkers from 22 patients found that CDK4 expression above the median had 6-month PFS compared to only 2-months in those with low CDK4 expression.	[73]
Therapeutic vulnerability to PARPi, olaparib	Osteosarcoma cell lines and xenografts in NRG mice are treated with PARPi.	*RB* loss causes hypersensitivity to PARPi in osteosarcoma cell lines and mice grafts.	[74]
*RB* loss and CHK1, PLK1, and aurora kinase inhibitors	CRISPR screens in TNBC cell lines with RB alterations	Knudsen et al. have shown that *RB* loss can be exploited to make cells highly sensitive to drugs that inhibit CDC25, CHK1, or PLK1.	[75]

### 5.2. Impact of RB Status on Targeted Therapies

To understand how RB status influences therapy response, it is necessary to revisit its central role in cell-cycle regulation. RB normally restrains progression through the G1/S checkpoint until conditions are favorable. In cancer, this control is often bypassed through *RB* loss or hyperphosphorylation, E2F overexpression, or amplification of CDKs or cyclins, leading to proliferation and tumorigenesis. Because of the centrality of this pathway, its dysregulation has become an attractive therapeutic target. Notably, given the high frequency of cell-cycle alterations in sarcomas, the activity of CDK4/6i has been extensively explored in preclinical models [76].

Preclinical studies have established RB as a critical determinant of CDK4/6i efficacy; however, conflicting data from clinical trials in breast cancer has failed to find definitive biomarkers, such as RB, *CDKN2A*, cyclin, or CDK status, highlighting the need for further investigation [71,72,77,78]. More recently, phase II study PalboSarc (NCT03242382) found palbociclib as an effective treatment in several sarcoma subtypes selected on the basis of biomarkers such as elevated CDK4 expression or *CDKN2A* loss [73]. Because clinical trials using targeted agents as monotherapies have shown limited long-term benefit, preclinical evidence recommends that combination approaches be investigated to prevent resistance emergence and increase efficacy using lower doses.

While the use of CDK4/6i in combination regimens is still emerging, preclinical evidence suggests that dual targeting with other agents may enhance their therapeutic efficacy. For example, in glioblastoma, where 80% of cases have RB/E2F pathway dysregulation, dual CDK4/6 and mTOR inhibition showed efficacy in both in vitro and in mouse xenograft models, by modulating cellular metabolism and inducing prominent apoptosis [79]. Additionally, emerging evidence indicates that RB-deficient osteosarcoma cells remain responsive to dual CDK4/6 and BETi by inducing DNA damage and apoptosis, highlighting the importance of investigating therapeutic strategies in RB dysregulated cancers [80]. An extensive review of the current progress of CDK4/6i-based combination strategies, including combinations with chemotherapy, endocrine therapy, PARP, EGFR, PI3K, CK1, WEE1, and immune checkpoint inhibitors, has already been published [81]. However, few studies have investigated the utility of these drugs in RB-deficient models. Such studies are needed to better understand the role of RB in cancer and identify combination strategies that may be effective in various subtypes, including those with RB dysregulations.

Mechanistically, *RB* loss is associated with dysregulated E2F activity and uncontrolled S-phase entry. This acceleration through checkpoints increases replication stress and genomic instability. Unlike other tumor suppressors, like P53, that function in response to stress or damage by inducing cell-cycle arrest, RB acts upstream as a regulator of cell-cycle progression. Consequently, RB dysfunction can directly promote replication stress through uncontrolled replication initiation, rather than responding to damage after it has occurred. Thus, RB-deficient cells become more dependent on DNA damage response and checkpoint pathways for survival.

This creates a therapeutic vulnerability that can be exploited through synthetic lethality approaches. For instance, CHK1 inhibition disrupts replication stress checkpoints, leading to replication fork stalling and collapse. Similarly, PARPi impair DNA repair processes, which, when combined with RB-associated defects in genomic stability, promote accumulation of DNA damage and apoptosis. These findings highlight replication stress dependency as a key mechanism underlying sensitivity to targeted therapies. Consistent with these mechanisms, growing evidence suggests that cancers with RB deficiency may still respond to therapy and, as such, should not be categorically excluded from treatment. Although research in this area is limited, these findings highlight the importance of identifying RB-dependent vulnerabilities and developing rational combination strategies within RB-deficient contexts.

Notably, a few reports have demonstrated therapeutic efficacy of targeted agents in RB-deficient cancers. For example, Zoumpoulidou et al. found that RB mutant cell lines and xenografts exhibited marked sensitivity to PARPi, comparable to the efficacy seen in BRCA1 and BRCA2 mutant cancers [74]. Similarly, Knudsen et al. found that, while *RB* loss accelerates tumor proliferation and is associated with CDK4/6i resistance, TNBC cells lacking RB were highly susceptible to CDC25, CHK1, and PLK1 inhibition through multiple cell-cycle control pathways [75]. Specifically, CDC25 inhibition prevented CDK activation and induced cell death, CHK1 inhibition exacerbated DSB and replication stress, and PLK1 inhibition promoted mitotic catastrophe. These findings, as summarized in Section C of Table 1, suggest that, although *RB* loss has historically been viewed as a therapeutic barrier, it may instead serve as a cancer-specific vulnerability that can be exploited through targeted or combination therapies.

## 6. Conclusions and Future Directions

RB has traditionally been recognized for regulating cell-cycle progression. Growing evidence suggests that RB also regulates pathways involved in transcription, genomic stability, DNA repair, differentiation, stemness, and apoptosis. Consequently, *RB* loss may not only accelerate proliferation but can also reprogram broad cellular pathways that create exploitable vulnerabilities. Additionally, early preclinical models suggested functional RB is required for response to CDK4/6i. Clinical data, however, have not established RB status as a reliable predictor of sensitivity or resistance. Many patients develop resistance to CDK4/6i through RB-independent mechanisms, indicating that RB-deficient tumors may retain sensitivity, particularly when combination regimens are used.

Effective clinical use of RB as a biomarker remains limited due to inconsistent detection methodologies and the absence of standardized approaches. Malorni et al. developed gene expression signatures of *RB* loss, which can improve functional readouts and have been shown to correlate with prognosis and discriminate between sensitive and resistant lines [82]. Integrating these novel tools into clinical practice will allow for accurate stratification of patients to guide treatment selection. Moreover, clinical trials are essential to define how RB status influences therapy response. Prior to therapy exposure, comprehensive data collection is required for patient stratification. Samples should be obtained serially from patients receiving therapy over time to identify potential resistance emergence and opportunities for treatment modification. To assess long-term response and durability, studies may need both prospective and retrospective evaluation. Only a few clinical trials aim to link outcomes to patient-specific molecular characteristics and monitor response to therapy over time. The ComboMATCH trial (NCT05564377) is one which tests targeted therapies, like PARPi and CDK4/6i, in patients with advanced and metastatic solid tumors. Their goal is to identify specific alterations that can predict drug response. These studies are essential for evaluating RB as a biomarker of resistance and sensitivity.

Finally, *RB* loss creates a distinct therapeutic state marked by replication stress, chromosomal instability, and mitotic defects. Exploiting these tumor-specific vulnerabilities will likely require the utility of combination therapies and the use of prospective clinical trial designs. More research in the RB-deficient context is needed to define how *RB* loss reprograms cellular signaling pathways to create these targetable vulnerabilities. Additionally, RB may play important roles in processes relevant to metastatic progression, including cellular plasticity, invasion, and interactions with the tumor microenvironment. However, the role of RB in metastasis remains incompletely understood and warrants further investigation, particularly in the context of aggressive disease. By reframing *RB* loss from a binary therapy sensitivity marker to a broader mechanism for generating exploitable cellular vulnerabilities, precision oncology efforts can more effectively target aggressive and treatment-refractory cancers. Standardized assays of RB characterization, mechanistic understanding of RB-mediated reprogramming in various cancers and thoroughly designed clinical trials are essential to realize the full potential of RB. From a clinical perspective, reliance on RB status alone may be insufficient to guide CDK4/6i use, underscoring the need for more comprehensive biomarker strategies.

## Data Availability

No new data were created or analyzed in this study. Data sharing is not applicable.

## Abbreviations

The following abbreviations are used in this manuscript:

RB	Retinoblastoma
CDK	Cyclin dependent kinase
CDK4/6i	Cyclin dependent kinase 4/6 inhibitors
PARPi	Poly (ADP-ribose) polymerase inhibitors
CIN	Chromosomal instability
BETi	Bromodomain and extraterminal domain inhibitors
DOX	Doxorubicin
STS	Soft tissue sarcoma
PFS	Progression free survival
TNBC	Triple negative breast cancer
DREAM	Dimerization partner, RB-like, E2F, and the multi-vulval class B
HDACs	Histone deacetylases
CHR	Cell-cycle gene homology regions
ESC	Embryonic stem cell
CSC	Cancer stem cells
EMT	Epithelial-mesenchymal transition
DSB	Double-strand breaks
NHEJ	Non-homologous end joining
HR	Homologous recombination
ER	Estrogen receptor
5-FU	5-Fluorouracil
CRISPR	Clustered regularly interspaced short palindromic repeats
